# Phenotypic Characterizations and Comparison of Adult Dental Stem Cells with Adipose-Derived Stem Cells

**Published:** 2010

**Authors:** Razieh Alipour, Farzaneh Sadeghi, Batool Hashemi-Beni, Sayyed Hamid Zarkesh-Esfahani, Fariba Heydari, Sayyed Behrouz Mousavi, Minoo Adib, Manizheh Narimani, Nafiseh Esmaeili

**Affiliations:** 1 Department of Immunology, Medical School, Isfahan University of Medical Sciences, Isfahan, Iran; 2 Department of Anatomical Sciences, Medical School, Isfahan University of Medical Sciences, Isfahan, Iran; 3 Department of Anatomical Sciences, Medical School, Isfahan University of Medical Sciences, Isfahan, Iran; 4 Department of Immunology, Medical School, Isfahan University of Medical Sciences, Isfahan, Iran; 5 Torabinegad Research Center, Dental School, Isfahan University of Medical Sciences, Isfahan, Iran; 6 Central lab, Medical School, Isfahan University of Medical Sciences, Isfahan, Iran; 7 Department of Immunology, Medical School, Isfahan University of Medical Sciences, Isfahan, Iran

**Keywords:** Stem cells, Prevention, Dental pulp, Mesenchymal, CD markers

## Abstract

**Objectives:**

Mesenchymal stem cells or “multipotent stromal cells” are heterogeneous cell population with self-renewal and multilinage differentiation. The aim of this study was to examine and compare the expression of important stem cell surface markers on two populations of mesenchymal stem cells, one derived from human exfoliated deciduous teeth and the other derived from human adipose tissue. These new stem cells will offer a promising avenue for prevention and reversal of many human diseases such as type 1 diabetes and prevention of liver fibrotic process.

**Methods:**

Mesenchymal stem cells were isolated and cultured from human adipose tissue and dental pulp of human exfoliated deciduous teeth. The cultured cells then were harvested and stained by different fluorescent labeled monoclonal antibodies against surface markers and were analyzed using flow cytometry.

**Results:**

Both different cell populations expressed CD44, CD90 and CD13 (stem cell markers) with similar intensity. They did not express hematopoietic markers (CD11b, CD19 and CD34), and lymphocyte or leukocyte antigens CD3, CD7, CD20, CD14, CD45, CCR5 (CD195), CD11b and CD10 on their surfaces. Two different cell types demonstrated different levels of expression in CD56 and CD146. Mesenchymal stem cells from human exfoliated deciduous teeth were positive for CD105 and were negative for CCR3 and CCR4 expression.

**Conclusions:**

Both cell populations derived from adipose tissue and dental pulp showed common phenotypic markers of mesenchymal stem cells. In conclusion, mesenchymal stem cells could be isolated and cultured successfully from dental pulp of human exfoliated deciduous teeth, they are very good candidates for treatment and prevention of human diseases.

## INTRODUCTION

About fifty years ago, Alexander Friedenstein et al. reported presence of a population of nonhematopoietic cells that were capable of autorenovation and bone differentiation in the bone marrow.^1^ Subsequently, others showed the bone-marrow-derived cells isolated according to Friedenstein’s technique, also possessed high potency of proliferation and pluripotency of differentiation into mesenchymal tissues, and therefore Caplan used the term “mesenchymal stem cell” (MSC) to describe them.^1^,^2^ Further studies have established mesenchymal stem cells as a heterogeneous cell population in which each individual cell varies in its gene expression, differentiative capacity, expansion potential and phenotype. Moreover, all of them do not seem to fulfill the stem cell criteria. Therefore, they are preferred to be called “multipotent stromal cell” with the same acronym “MSC”.^1^,^3^ While MSC were traditionally obtained from bone marrow, later, MSC-like cells isolated from a variety of human tissues including muscle connective tissue, perichondrium, adipose tissue, peripheral blood, dental pulp and also fetal tissues such as lung, liver, spleen as well as from amniotic fluid, placenta and umbilical cord blood (UCB).^3^,^4^ At present, any cell population which meets the following characteristics, irrespective of its tissue source, is generally referred as MSC: morphologically, they adhere to plastic and have a fibroblastlike appearance; functionally, they have the ability of self-renewal and could differentiate into cells of the mesenchymal lineage (osteocyte, chondrocyte and adipocyte), also into cells of the endoderm (hepatocytes) and ectoderm (neurons) lineages under proper cell culture conditions; phenotypically, they express a set of almost given markers and do not express another set of known markers that will be discussed later.^5^,^6^ In the nomenclature of stem cell biology, MSC are classified as pluripotent, adult stem cells that can be easily and without serious moral obstacles (unlike the embryonic stem cells) isolated from various tissues and simply expanded *ex vivo*.^2^,^6^ Therefore, they have been at the center of attention for the use in the cell therapy, regenerative medicine and tissue engineering. After all, mesenchymal stem cells have the capacity to suppress the activation of immune cells and reduce the inflammation.^7^,^8^ In other words, MSC have the unique immunoregulatory ability which extends their clinical application to transplantation and autoimmune diseases. The immunomodulatory effects of MSC may be used to restore tissue damage caused by inflammatory diseases such as multiple sclerosis, inflammatory bowel disease and rheumatoid arthritis in humans, as well.^7^’^9^ During solid organ transplantation, to prevent transplant rejection, the individual has to be under a life-long non-specific immunosuppressive therapy that may tend to lots of complications. It has been shown in variety of animal models, that using MSC could prevent rejection and induce prolong graft acceptance or at least minimize dependency on long-term use of immunosuppressive drugs following the transplantation; consequently, prevent the significant side effects of immunosuppressive drugs’ administration such as drug toxicity, opportunistic infections and malignancies.^10^,^11^ The most promising results of clinical application of MSC have been observed in bone marrow (BM) transplantation; here they not only prevent and ameliorate graft-versus-host disease (GVHD), but also increase BM engraftment and improve its function.^2^,^3^ These results have been encouraging enough that scientists started clinical trials in human. Double-blind, randomized studies are under way using MSC versus placebo in Europe and the USA to investigate the effect of MSC on GVHD and enhancing engraftment in patients undergoing hematopoietic stem cell transplantation (HSCT).^9^,^12^ MSC transplantation has been successfully used for prevention of progressive heart dysfunction after myocardial infarction.^13^ Mesenchymal stem cell transplantation has been proved beneficial in the prevention of liver fibrotic process.^14^ Despite lots of encouraging data on MSC applications in the different clinical setting and the vast quantity of experiments focused on MSC, several open questions have remained about their biology; of the important ones is their phenotype. To date, a large number of studies have been conducted to identify the markers expressed by MSC. Unfortunately they have failed to result in identification of exclusive markers or even a specific collection of markers for MSC that could be used for identification and isolation purposes.^6^,^9^ Over the recent years, a variety of phenotypic markers including adhesion molecule, lineage antigens, growth factor receptors, cytokine/chemokine receptors, immune-related proteins etc, on MSC from different origins, have been investigated in many researches. Conflicting results emphasize the need for gathering more information to complete our understanding of MSC phenotype.^15^’^17^ Although the rang of the considered cell surface markers is substantially wide, nevertheless a general overlook of them, infers that MSC derived from multitissues and organs have some phenotypes in common. Among the markers have been more consistently reported, the Mesenchymal and Tissue Stem Cell Committee of the International Society for Cellular Therapy (ISCT) have established a certain panel of cell-surface markers including the followings: about 95% of the MSC population must express CD73, CD90 and CD105, and no more than 2% of the cells express CD34, CD45, CD11b or CD14, CD19 or CD79a and HLA-DR. The phenotype may undergo further modification, since this field is still under investigation.^18^,^19^ Regarding the ongoing discussion about MSC phenotype, we examined and compared the expression of about twenty surface markers, using dual color flow cytometry technique, on two MSC populations derived from two distinct tissue sources, MSC obtained from human adipose tissue (ADSC) and stem cells from human exfoliated deciduous teeth (SHED). Among the markers analyzed in the present study, some of them exist in the list provided by ISCT as positive (CD90 and CD105) or negative (CD34, CD45, CD11b, CD14, CD19 and HLA-DR,) markers; another set of the antigens such as CD44, CD146, CD25, CD33, CD13, CD3, CD56, CD16, CD10, CCR3, CCR4, and CD195 have been examined by other studies on MSC. The expression of CD66b was also tested on MSC for the first time in this study.

## METHODS

### 

#### MSC cell culture

ADSCs were isolated and cultured as reported previously.^20^ In brief, the sample removed from subcutaneous adipose tissue of a woman as waste product during surgery at AlZahra University Hospital (Isfahan, Iran) and rapidly transported to laboratory in cold sterile phosphate buffer saline (PBS). After washing 3 times with PBS, it was weighted and unwanted connective tissues and vessels were cut away and simultaneously, the adipose tissue minced with a scalpel knife. Thereafter, it was digested with collagenase IA (Sigma Aldrich), at 37°C for 30 minutes. After incubation, the enzyme was neutralized by adding complete cell culture medium (DEMEM medium supplemented with 10% FCS and 1% penicillin/streptomycin, Gibco), the mixture was centrifuged and the pellet was cultured in complete cell culture medium. SHED were isolated as instructed by Huang et al.^21^ The extracted pulp tissues from normal exfoliated deciduous teeth of six- to nine-year-old children digested in collagenase type I (Sigma Aldrich) for 1 hour at 37°C. The harvested cell suspensions were filtered through a 40 μm cell strainer and the single cell suspensions were cultured in DMEM medium supplemented with 10% FCS at 37°C with 5% CO2. After two to three days, when the cells reached optimal confluency (70 to 90 percent), they were trypsinized and collected for further passages.

#### Cell phenotype determination

Both SHED and ADSC cells at passage 4 were used for flow cytometric analysis. The cells were incubated with either FITC or PE labeled monoclonal antibodies (mAb) according to the manufacturers’ protocols. Briefly, one 75 cm^2^ flask of the cells were trypsinized, washed with PBS containing 10% BSA (washing buffer) and resuspended in small volume of washing buffer. The harvested cells were counted and the volume was adjusted using washing buffer to give a cell density of 10^6^cells/ml. Then, 100 μl of cell suspension (about 10^5^ cells) were transferred into each FACS tube and appropriate amount of antibody was added and incubated at room temperature (RT) for 30 to 45 minutes in a dark place. The used antibodies were as follows: FITC-labeled mAb against human CD90, CD10, CD7, CD3, CD56 (all from IQProduct), CD66b, CD11b (both from Serotec), CD45 (LeucoGATE), CD25, CD34 (both from BD Pharmigen), and PE-labeled mAb against human CD44, CD13, CD20, CD22, CD33, CD16, CD19, HLA-DR (all from IQProduct), CD14 (LeucoGATE), CD146, CCR3, CCR4, CCR5 (all from BD Pharmigen) as well as unlabeled mAb against human CD105 (Abcam). Appropriate fluorescent labeled isotype matched control antibodies were also used. After incubation, the cells were washed with washing buffer twice. In the case of CD105, an extra incubation period with a secondary mAb (goat FITC-labeled against mouse Abs (Serotec) followed by washing was also performed. Finally, cells were examined on a FACSCalibur flow cytometry (BD Biosciences) using CellQuest data acquisition and analysis software. MSC were located using FSC, SSC parameters and a live analysis gate was set around this population. Data were acquired from 10,000 cells (events) and the proportion of given cell populations expressing the desired marker was determined.

## RESULTS

**Figure 1:**
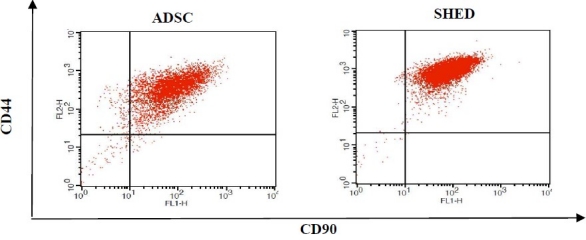
Both mesenchymal populations showed similar shape in the cell culture. They were adherent cells with spindle-shaped, fibroblast-like appearance

**Figure 2:**
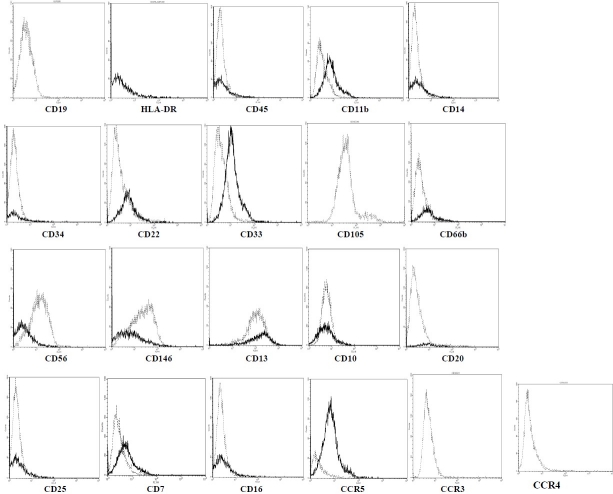
CD marker expression of two MSC populations, ADSC and SHED. The cells were incubated with fluorescent labeled antibodies against the indicated antigens and analyzed by flow cytometer. The histograms of two MSC types were overlaid. The histograms corresponding to ADSC are demonstrated by bold line and those corresponding to SHED are shown by spotted line.

ADSC and SHED were cultured as described in materials and methods section. These cells exhibited the expected fibroblast like morphology (Figure 1). To further ensure the stemness characterization of isolated MSC cells, the capacity of both ADSC and SHED cells to differentiate into chondrocyte and osteocyte were also assessed (data not shown). Flow cytometric analysis showed that cells were expressing the phenotypic markers shown in Figure 2. Both the ADSC and SHED were negative for CD34, CD14, CD45, CD11b, CD3, CD10, CD20, CD33, CD22, CD16, CD7, CD25, CD33 and CCR5. SHED also were negative for CD19, CCR3 and CCR4 as well as ADSC for HLA-DR expression. ADSC and SHED, both expressed CD44 and CD90 at high level and CD13 at low level. CD56 and CD146 were detected only on SHED. CD105 expression was observed on SHED as well.

## DISCUSSION

Mesenchymal stem cells or more truly multipotent stromal cells have some properties which give them many attractive clinical potential applications such as prevention of human diseases including prevention of progressive renal failure.^22^ They could be obtained from many adult and fetal tissues. They should be wellcharacterized before they could be used for treatment. One of the unsolved problems in the case of MSC is their phenotypes. So far, despite extensive investigations, scientists have not been able to reach a global consensus about that. Considering this issue, and the need for new sources of feasibly available stem cells, we isolated adult stem cells from two different sources (ADSC and SHED) and determined expression of about 20 important surface markers on these two populations. ADSCs are multipotent stromal cells derived from adipose tissues. Adipose tissue is one of the first tissues used as source of MSC and because of its availability and simple MSC isolation procedure from that, they have been the subject of many studies. SHED or stem cells from human exfoliated deciduous teeth are rather newly discovered MSC^23^ that are classified as Dental MSC which are derived from the ectomesenchyme.^24^ They are more potent in proliferation than bone marrow derived MSC (BM-MSC) and interestingly express many of neural cell markers in the cell culture.^23^,^24^ However, compared to ADSCs they are less-studied and information about their biology and phenotypic markers are less available. Expressions of cell surface markers were studied using flow cytometry. Both cell types expressed CD90 and CD44 (cell adhesion receptor) at high levels. They did not express CD45 (common leukocyte antigen), CD14 (monocyte and granulocyte marker), CD11b, CD19, CD34 (hematopoietic cell lineage marker) and CD3 (lymphocyte antigen). In addition, SHED expressed CD105 (Endoglin, SH2). Therefore, both cell populations showed the current usual phenotypic markers of MSC. CD22 and CD33 were neither expressed on SHED nor on ADSC. These results are generally in agreement with others reported earlier.^25^,^26^ CD22 and CD33 are two well-known molecules in Siglect (the sialic-acid-binding immunoglobulin-like lectins) family of receptors and generally have restricted expression on leukocytes and other immune cells.^27^,^28^ CD66, a marker which is analyzed in few studies on MSC were negative for both of our cell populations. Although, there are reports of its expression on MSC derived from bone marrow^29^,^30^ and initially, it seems in conflicting to our results, it is notable that CD66 comprise a group of 12 glycoproteins that except one, all are cell surface molecules. They also are termed the carcinoembryonic antigen (CEA) related cell adhesion molecule (CEACAM) family. The member of the family distinguished by numbers; traditionally CEACAM-1, CEACAM-3, CEACAM-5, CEACAM-6 and CEACAM-8, designated as CD66a, CD66d, CD66e, CD66c and CD66b, respectively. Different members of the CEACAM family have different special activities as well as their own expression patterns on normal and cancer tissues.^31^,^32^ Ghazanfari et al. and Shin et al. reported that the CD66 is a positive marker for MSC.^29^ The exact type of CD66 was not determined and they used antibodies against CD66 that identify antigens from this CEACAM family of proteins, but we used antibody against CD66b. Therefore, it is possible that only BM-MSC expresses this antigens and other member of CEACAM family, rather than CD66b, is expressed on SHED or ADSCs. As CD66b is exclusively expressed on human granulocytes and it is recognized as a granulocyte “activation marker”,^33^ our results could be justified. The expression of CD146 was different in ADSC and SHED. While SHED was positive for this marker, we were not able to detect that on ADSCs. CD146 or Mel-CAM, MCAM or MUC18, is a highly glycosylated transmembrane glycoprotein part of the immunoglobulin (Ig) superfamily, initially recognized in melanoma cells. CD146 expression has been determined in normal mature tissues, such as vascular endothelium and smooth muscle, also in a subpopulation of activated T cells. On the other hand, it is expressed during the stages of embryonic development. However, CD146 is considered as an adhesion marker of endothelial cells, since it has been used to identify and isolate these cells from peripheral blood. Interestingly, CD146 has been recently known as a MSC marker.^34^ Its expression has been shown on MSC from different origins including ADSCs^35^ and particularly MSC.^25^,^36^ CD146 has been even applied efficiently for isolating populations of human MSC *in vivo*. It is thought that its expression is related to probable perivascular nich of MSC.^37^ Our results showed the expression of CD146 on SHED, but not on ADSC. However, our negative results about the absence of CD146 on ADSCs is not the only report in this regard as others have reported similar findings.^38^,^39^ This can be explained by the heterogeneity of MSC, and probably there are subpopulations of MSC that do not express CD146. CD56 was the other marker which its expression was different in two MSC populations. In the present study, SHED expressed this molecule, but ADSC were negative for that. Although many investigators could not detect CD56 on MSC,^40^,^41^ recently Battula et al. reported isolation of a distinct mesenchymal stem cell subset in BM that expressed CD56 on their cell surfaces.^42^ This finding can explain our results, however they used a mAb against CD56 which recognized an epitope that does not express on natural killer (NK) cells,^43^ while we used mAb against CD56 that has been applied before for identifying NK cells. It may mean that SHED has the same CD56 molecule as NK cells, which is different to CD56 which Battula et al. detected on BM-MSC.^42^ CD7, CD20, CD25 and CD16 were absent on both cell types which is in agreement with what have been reported before.^26^,^40^,^44^,^45^ It has been reported by many researchers that MSC, in particular dental MSC, express CD13 on their surfaces^20^,^35^,^46^,^47^ while there are few reports that were not able to detect CD13 on MSC.^26^ In our study, both MSC groups were positive for this surface molecule. The data about CD10 expression on MSC is more confusing compared to CD13.^20^,^48^ Even two groups of MSC obtained by Battula et al. from BM were different in CD10 expression.^42^ We also were not able to detect CD10 on ADSC and SHED. A number of studies have confirmed that MSC respond to some cytokines and chemokines through the expression of their receptors on their own surfaces. For example, CCR4, CCR7, CCR9, CXCR4, CXCR5 and CXCR6 are some chemokine receptors that have been reported to be present on MSC. It is now generally accepted that MSC can respond to inflammation by their CXCR4 receptors.^15^,^17^ In this work, we demonstrated that CCR3 is not present on either SHED or ADSC. CCR3 is a chemokine receptor for CCL chemokines that generally causes the immune cells (monocytes/macrophages, T cells, eosinophils, basophils, NK cells and dendritic cells) to respond to inflammatory chemokines MIP-1α and MIP-1β or other cytokines such as RANTES and eotaxin. Honczarenko et al. and Ponte et al. reported the expression of CCR4 and CCR3 on MSC^48^,^49^ that seems in contrast to our results, but the striking point is that they all worked on BM-MSC, so maybe it is reasonable to relate this controversy to potential different expression pattern of chemokine receptors on diverse population of MSC. In conclusion, mesenchymal stem cells could be isolated successfully from both adipose tissue and tooth. These cells have great potential to be used for treatment and prevention of many human diseases.
